# How Does Our Knowledge on the Na^+^/H^+^ Exchanger NHE1 Obtained by Biochemical and Molecular Analyses Keep up With Its Recent Structure Determination?

**DOI:** 10.3389/fphys.2022.907587

**Published:** 2022-07-15

**Authors:** Mallorie Poet, Denis Doyen, Emmanuel Van Obberghen, Gisèle Jarretou, Yann Bouret, Laurent Counillon

**Affiliations:** ^1^ Université Côte d’Azur, CNRS, Laboratoire de PhysioMédecine Moléculaire (LP2M), Nice, France; ^2^ Laboratories of Excellence Ion Channel Science and Therapeutics, Nice, France; ^3^ Centre Hospitalier Universitaire de Nice, Nice, France; ^4^ Université Côte d’Azur, CNRS, Institut de Physique de Nice (INPHYNI), Valbonne, France

**Keywords:** Na^+^/H^+^ exchanger 1, structure function studies, allosteric regulation, kinetics, protein 3D structure

## Abstract

Na^+^/H^+^ exchangers are membrane transporters conserved in all living systems and therefore are assumed to be amongst the most ancestral molecular devices that equipped the first protocells. Following the cloning and sequencing of its gene, the mammalian NHE1, that regulates pH and volume in all cells, has been thoroughly scrutinized by molecular and biochemical analyses. Those gave a series of crucial clues concerning its topology, dimeric organization, pharmacological profile, regulation, and the role of key amino acids. Recently thanks to cryogenic Electron Microscopy (Cryo-EM) the long-awaited molecular structures have been revealed. With this information in mind we will challenge the robustness of the earlier conclusions and highlight how the new information enriches our understanding of this key cellular player. At the mechanistic level, we will pinpoint how the NHE1 3D structures reveal that the previously identified amino acids and regions are organized to coordinate transported cations, and shape the allosteric transition that makes NHE1 able to sense intracellular pH and be regulated by signaling pathways.

## Introduction

The Na^+^/H^+^ exchangers of the SLC9A gene family (NHEs) are expressed in all mammalian cells and tissues where they exert multiple physiological roles and share functional redundancy with other membrane transport proteins (Doyen et al., 2022). While NHE1 mostly regulates pH and cell volume, the epithelial NHE2, 3, 4 are important for mediating salt and bicarbonate balance across epithelia such as kidney and intestine while NHE8 is crucial for intestine goblet cells mucus secretion (for review see Aronson et al., 1982; Pedersen and Counillon 2019). The vesicular NHEs (6, 7, and 9) regulate pH in intracellular compartments. Several NHEs have been implicated in disease situations, that can be linked to NHE mutations, su1993bch as for example, the Christianson’s syndrome for NHE6 (Gilfillan et al., 2008) that includes both neurodevelopmental and neurodegenerative defects, the autism spectrum disorders for NHE7 and NHE9 (Morrow et al., 2008), or chronic diarrhea (Schultheis et al., 1998). NHEs are also involved in acquired pathologies such as heart ischemia-reperfusion for NHE1 (Lazdunski et al., 1985, for review see for example; Pedersen and Counillon 2019).

Briefly, the mammalian Na^+^/H^+^ exchange activity was measured for the first time by Heini Murer and collaborators (Murer et al., 1976) and the first Na^+^/H^+^ exchanger cDNA encoding NHE1 was cloned a decade later by the Pouysségur’s group (Sardet et al., 1989). This was followed by the cloning of the other NHE2-9 isoforms expressed notably in epithelia or intracellular compartments (Tse et al., 1992; Tse et al., 1993; Orlowski et al., 1992; Attaphitaya et al., 1999; Numata and Orlowski 2001). The sequences of these exchangers led to the production of hydropathy plots that gave the first insight into the NHEs as membrane proteins, as well as sequence comparison that enabled to identify highly conserved amino acids possibly involved in transport or regulatory functions and hence pointing to conceivable pharmacological interventions. Thanks to these cDNAs and the selection of NHE-deficient cell lines, PS120 cells (Pouysségur et al., 1984), and AP Cells (Rotin and Grinstein 1989), this also allowed the application of somatic cell genetics and site directed mutagenesis to study the relations between NHE’s sequence, topology and function. This generated a whole set of key observations but an unified transport mechanism could not be presented in the absence of any structural data, because of the risk of missing or misinterpreting important information and of reliably connecting them. We will see in this review that kinetic schemes are also required to ensure a suitable treatment of mutagenesis data. In this context a long-awaited breakthrough in the field has been the nearly concomitant resolution of the structure of NHE1 and NHE9 by Cryo-EM (Dong et al., 2021) and (Winklemann et al., 2020). Both articles and their supplementary information highlight for these transporters their chief structural information, functional sites and mechanisms of regulation.

Hence, the purpose of our review, within this dedicated special issue on the Forever Young Na^+^/H^+^ exchanger, is not to recapitulate the information given in these articles but rather to revisit the main questions and findings that had intrigued the scientific community in the last 3 decades. In particular, we will evaluate to which extent the earlier findings stood the test of time or were forced to be re-conceptualized now that they can be refigured in the recently obtained structures. We will also highlight how biochemical, molecular and structural analysis have mutually enriched the efforts to decipher the particularly important physiological mechanism of proton sensing by NHEs.

For the sake of space and clarity, this review article will mostly focus on the ubiquitous NHE1 that has been subjected to most of the structure-function work. For this transporter, the paper by Dong et al. reports three Cryo-EM structures, 1) one NHE1 CHP1 complex obtained at pH6.5, 2) one generated at pH7.5 that is not noticeably different from the previous one but contains less information on the CHP1 interaction, and 3) one cariporide bound CHP1complex that is slightly more open and probably stabilized as an outward-facing high affinity for H^+^ form. Considering the three models we will mostly compare the pH6.5 and cariporide structures. Of note, the CHP1-NHE1 Cryo-EM structures are in good accordance with the previous structure of CHP1-with a part of NHE1 intracellular loop obtained by Wakabayashi and colleagues (Ammar et al., 2006; Mishima et al., 2007).

## Topology and Structural Organization of NHE1

The first cDNA encoding a mammalian Na^+^/H^+^ exchanger was cloned using genetic complementation and revealed from the start the arrangement characteristic of a transmembrane protein, with 10–12 hydrophobic stretches identified by hydropathy plots as putative transmembrane segments (Sardet et al., 1989).

This topological model predicted a protein with both a N and C terminal inside with a protein globally divided into two large regions: a ∼500 amino acids long transmembrane part that was subsequently shown to be necessary and sufficient for transport (Wakabayashi et al., 1992) and a large intracellular tail with multiple sites for regulatory proteins as well as intrinsically disordered regions (Hendus-Altenburger et al., 2017).

The global NHE1 transmembrane organization was subsequently confirmed by the following three observations: 1) the antibody accessibility against the intracellular loop requiring cell membrane permeabilization (Sardet Science 1990), 2) the identification of glycosylation sites on the extracellular loop 1 (Counillon et al., 1994; Tse et al., 1994), and 3) the analysis of protease accessibility assays (Shrode et al., 1998).

Subsequently, Shigeo Wakabayashi’s group used the substituted cysteine accessibility method (Karlin and Akabas, 1998) to dissect the positioning of the different amino acids and loops (Wakabayashi et al., 2000). This yielded the first experimentally refined topological model of NHE1 that served as widely accepted reference for exploring the NHEs structure for the next 20 years, until the Cryo-EM structures of NHE1 and NHE9 were finally published. Strikingly this topology was also in good accordance with sequence alignment of NHEs across multiple species. See for example the multiple alignments of Na^+^/H^+^ exchangers sequences (Pfam0099) in the Pfam database of protein families (https://pfam.xfam.org).

This is likely explained by the fact that most of the amino acids in the transmembrane segments of a protein are much more conserved than in loops because they can be involved either in the protein structure, or in the transport mechanism, or in both. In contrast, loops are much more flexible allowing for more sequence variation, with of course some very conserved positions for important amino acids (Pedersen and Counillon 2019).

**FIGURE 1:**
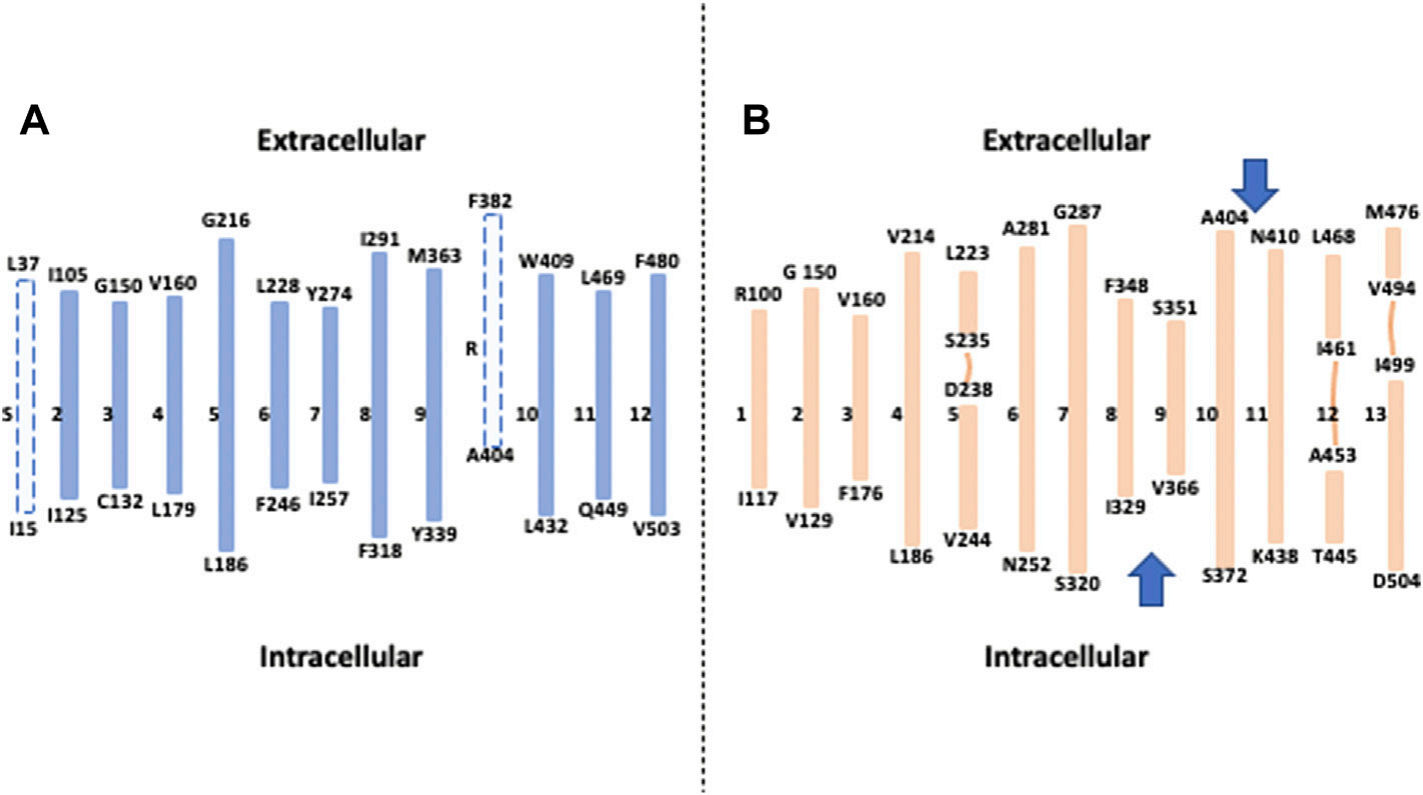
How the Cryo-EM structure changed our view of NHE1 topology: **(A)** Topological model deduced from hydropathy plots, sequence conservation and cysteine accessibility assays. Dotted lines represent respectively a putative signal peptide (S) and a potential transmembrane segment that was subsequently described as a re-entering loop(R). **(B)** The different transmembrane segments obtained from the NHE1 structure at pH 6.5. The lines represent interrupted helices, and the two arrows show topological inversions compared to the model in A.

When unfolded in transmembrane segments, the overall structure for NHE1 reveals a generally satisfactory accordance with the previous topology models together with some interesting discrepancies relating to structurally and functionally important features (Figure 1). When excluding the first very short putative TM that was very early on supposed to be a cleaved signal peptide and does not appear on the Cryo-EM structures, all the TMs are in excellent accordance with the actual TM 1 to TM8, where a first topological inversion occurs (Figure 1B). This persists until the actual TM10 that was considered as a reentering loop in the previous topological models, while some hydropathy plots had envisioned it as a putative transmembrane segment (Figure 1A) (Wakabayashi et al., 2000). This results in a second topological inversion that enables to have the two last transmembrane segments in the same orientation as in the Wakabayashi’s model, with finally the C terminal regulatory region in the cytosol as expected. When zooming on the transmembrane segments themselves, one notable difference is the actual boundaries of the helices between the models and the structures. While transmembrane segments have been mostly predicted to start at hydrophobic residues to match the bilayer hydrophobicity, a significant part of the transmembrane segment’s boundaries contain polar or charged amino acids. This is potentially interesting in terms of future functional characterization as many polar or charged residues that were left unnoticed because in formerly predicted flexible loops are now localized at the edge of more rigid helices where they could intervene in ion coordination or in lipid polar headgroups interaction for example. Furthermore, when comparing the alpha helices between the pH6.5 and cariporide-bound conformations, we could observe that in addition to substantial movements, the transmembrane helices’ boundaries can be slightly different, suggesting some flexibility in hydrogen bonding at their edges. As we will see later, this is of particular importance for the allosteric transition that takes place at the dimer interface.

Taken together, the NHE model that has been used for more than 20 years has been accurately predicting 10 transmembrane segments out of 13, which is an impressive achievement. The main differences come from boundaries and stretches of sequences that were impossible to resolve given the information and experimental tools available.

**FIGURE 2:**
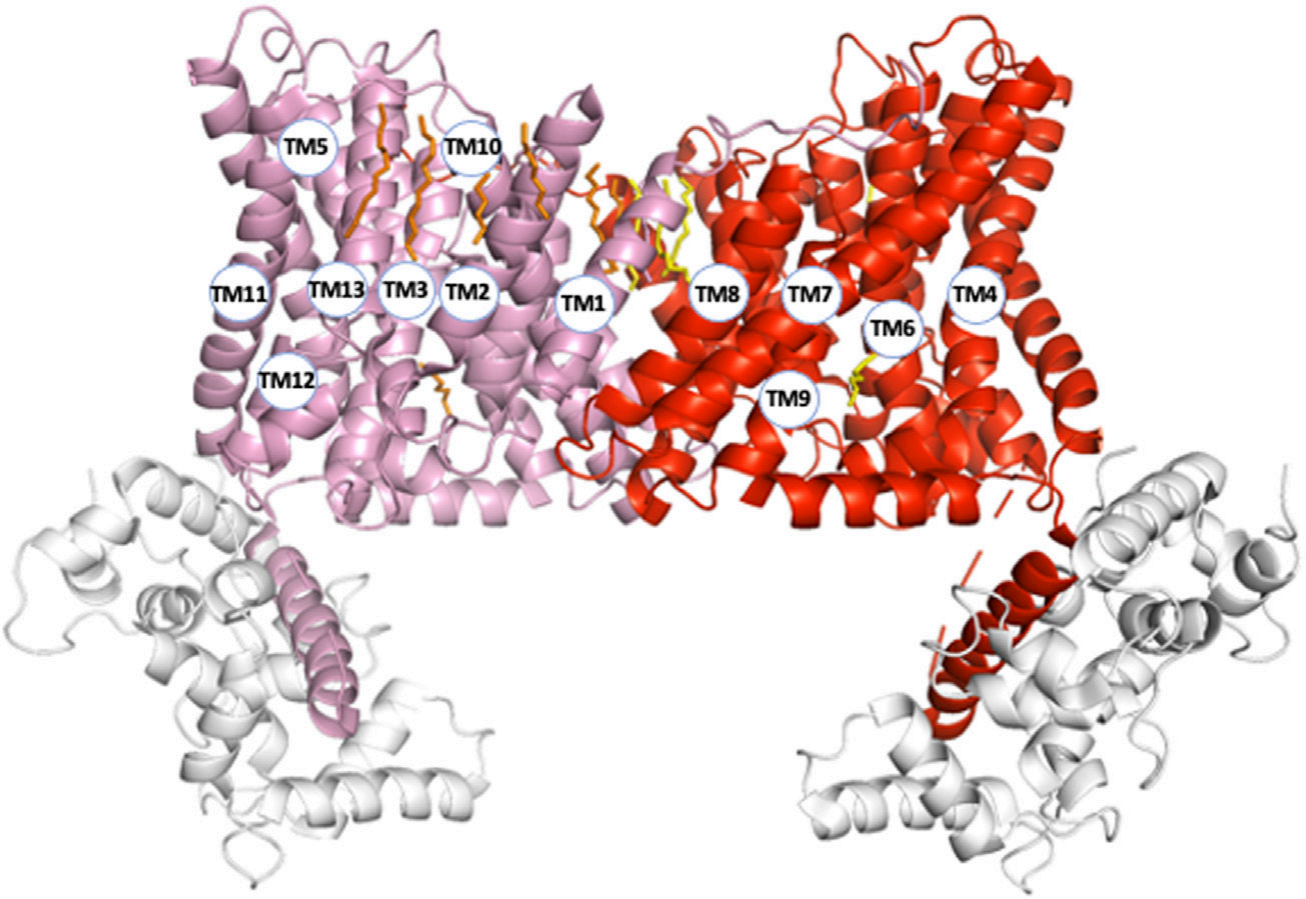
NHE1 structural organization (pH6.5). To visualize the organization of all transmembrane segments, the two protomers (respectively in pink and red) are represented within the symmetrical dimer. Numbers correspond to the transmembrane segments in Figure 1B. The CHP protein bound to NHE1 C-terminal region is represented in light grey.

Another remarkable feature is the high level of complexity of NHE1 structure, similar to those of many other transmembrane transporters. First the length of the transmembrane helices can vary considerably from simple to double for example, 17 amino acids for TM1 compared to 32 for TM10. These large differences fashion the overall shape of the NHE1 structure that is much thinner in the middle of the dimer, where it spans about 30 Å across the membrane plane, than on its side where it spans more than 50 Å, a geometry rendered possible by the large tilts of the longest helices (e.g., TM4, TM7, TM11) situated on the most external part of the structure (Figure 2). Such a geometry with tilted helices at the exterior and a comparatively smaller dimer interface could favor the mechanical coupling between the two protomers, because it would minimize the torque at the dimer interface. This could be important in the context of the NHE1 response to mechanical signals (Lacroix et al., 2008).

**FIGURE 3:**
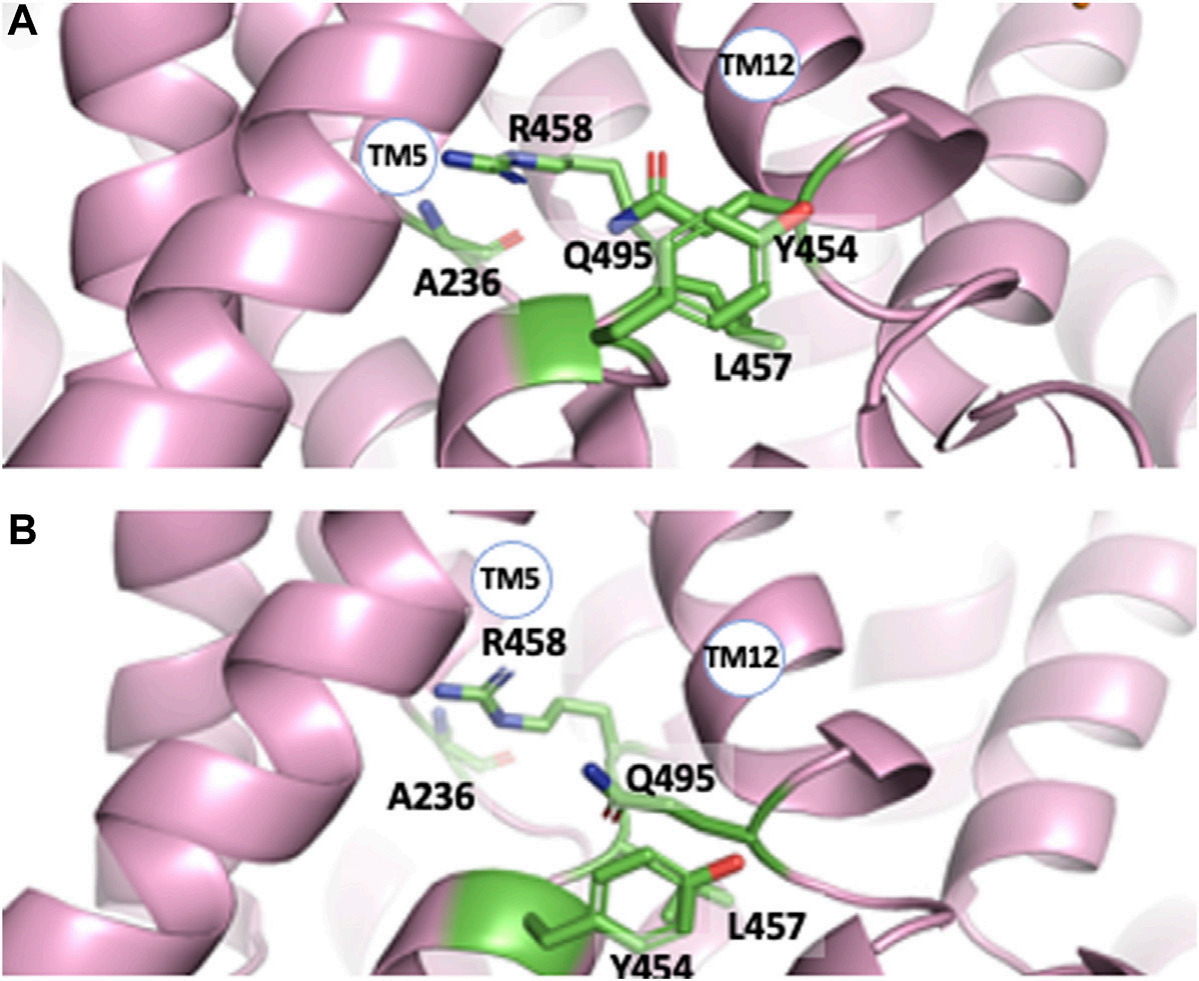
The interrupted transmembrane alpha helical segments 5, 11, and 12, in the pH 6.5 **(A)** and Amiloride-Bound structure **(B)** respectively. Note the close vicinity of these regions and the rotation of the Tyr 453-Leu 457 plane with respect to Gln495 between both structures.

All structures also reveal interrupted alpha helices, principally in transmembrane segments 5, 12, and 13 with some amino acids in these regions showing important degrees of conservation (Pedersen and Counillon 2019). Such flexible stretches, that leave also free carbonyls and amine hydrogens for possible interactions, are clustered in very close vicinity to each other in all Cryo-EM structures (Figure 3). Several amino acids could be involved in their interlocking, such as for example Arg 458 (TM12) and Ala 236 backbone carbonyl (TM5). Gln 495 (TM12) is crammed between Leu 457 and Y 454 of TM11 in the pH 6.5 structure (Figure 3A) and is slightly upwards form the plane of these two residues in the cariporide bound sequence (Figure 3B). Noticeably also, the large TM12 flexible loop crosses the protein middle section making this segment starting on the external surface of NHE1 and connecting to the last helix in the core of the transport region. This latter helix is immediately followed by the C terminal regulatory region, with its lipid and CHP binding sites. Taken together, the location of these internal flexible loops makes their roles worth investigating in future studies.

## The Dimer Interfaces

Following the production of the first anti NHE1 antibodies that enabled to visualize the protein in western blots (Sardet et al., 1990), it immediately became apparent that the transporter existed as a homodimer (Fafournoux et al., 1994). Its structure was disulfide bond independent, stable in the plasma membrane and to a certain extent SDS-resistant, the later point suggesting that hydrophobic helix-helix interactions in the transmembrane part maintained the dimer (Lemmon et al., 1992). In parallel we found that the dimeric structure of NHE1 was crucial for its regulation by intracellular pH, (Lacroix et al., 2004) a feature the importance of which will be discussed in a later chapter. The oligomeric nature of NHEs led to the identification by the Wakabyashi’s group of regions involved in this dimeric interaction (Hisamitsu et al., 2004; Hisamitsu et al., 2006). They used for a large part mutagenesis into cysteines followed by crosslinking, with an uncleavable bifunctional sulfhydryl reagent to map positions that would be in close vicinity within the dimer. Briefly, this allowed to identify positions 375 and 381 in NHE1 transmembrane domain. This was completed by the discovery that the deletion of a short stretch of sequence between Cys561 and Ala575 in the soluble cytosolic region had a negative effect on the dimer stability and allosteric coupling of NHE1. In contrast, mutating the upstream position 538 had no impact (Hisamitsu et al., 2004; Hisamitsu et al., 2006). The possibility that lipids, in particular PIP2, could be involved in the dimer stabilization cannot be excluded, as it has been found in the horse NHE9 dimer (Winklemann et al., 2020).

**FIGURE 4:**
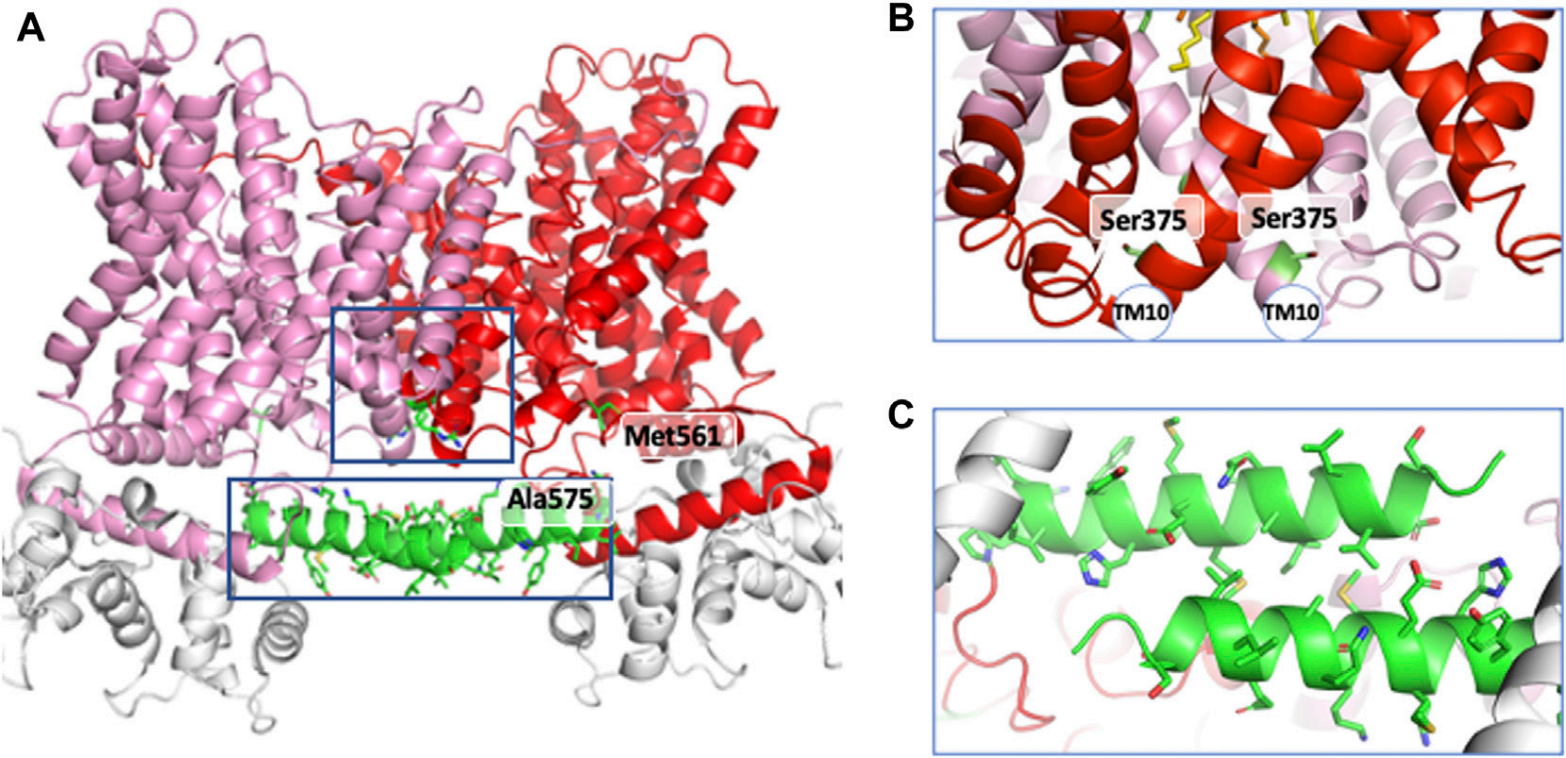
The helical regions involved in dimer interfaces. **(A)** in the general structure, **(B)** in the transmembrane region and **(C)** in the cytosolic loop of NHE1.

Examination of the NHE1 structure at the dimer interface within the transmembrane segment is in very good accordance with the previous findings. Indeed, it reveals a large plane of contact with strong helix-helix interactions, in particular between the 2TMs 11 that face each other close to Ser375, as well as TM8 and TM1 (Figures 4A,B). In the cariporide bound structure the Cys561-Ala575 stretch of sequence in the C terminal soluble tail is situated just before an amphipathic helix that starts at Pro571 and forms a beautiful antiparallel helix-helix interaction with its exact counterpart parallel to the membrane plane (Figures 4 A,C). Hence the structure clearly shows how the 561–575 deletion will destroy this interaction.

## Transporters Are Not Receptors: Kinetics and Structure Are Indispensable to Interpret Mutagenesis

Schematically any analysis of the relationship between the structure and function of NHE1 has to address the following key questions: 1) how and where do the extracellular Na^+^ and/or competitive inhibitors bind ? 2) is it possible to identify key kinetic steps, regions and conformations that provide clues to the translocation mechanism, and 3) how does a proton bind and regulate the rate of exchange, thereby enabling cells to control their intracellular pH ? Concerning the above-mentioned questions, we would like to stress that data obtained from studies using mutated transporters should be analyzed with caution in order to avoid erroneous conclusions. Therefore, in this review, we will draw attention to a series of key principles for the biochemical analysis of affinities and kinetics, which should help to correctly interpret the consequences of the mutations on the structure.

NHEs achieve both a kinetic and thermodynamic tour de force by 1) first allowing ions to cross the energy barrier provided by the hydrophobic membrane, and 2) secondly performing a coupled reaction that uses the energy stored in the Na^+^ transmembrane gradient to transport H^+^ ion against their electroosmotic gradient. In this context they can be considered as genuine enzymes that catalyze coupled reactions. Hence, it is important to realize that the mechanisms of transport can be formalized usefully using the conceptual and mathematical tools provided by enzyme kinetics as explained in the next section. Keeping this aspect in mind it helps to avoid mistakes that relate to fuzzy concepts called “affinity” for substrates or inhibitors, or “setpoints” or “sensing” for allosteric regulators.

### Na^+^ and Inhibitor Interaction Are Interlocked: The Michaelis-Menten Equation is Great But Does Not Make It all

Like we previously mentioned, NHEs enable extracellular Na^+^ to cross an energy activation barrier constituted by the hydrophobic bulk of membrane lipids and to flow according to its thermodynamic gradient between the outside and inside of cells. At steady-state, the shape of Na^+^ dose response curves for most of the NHEs can be approximated as a hyperbolic saturation function (Pedersen and Counillon 2019). Classically the Michaelis-Menten equation for a saturation curve with extracellular sodium can be written as

$$V=Vmax\frac{\left[Na_{e}^{+}\right]}{Km+\left[Na_{e}^{+}\right]}$$ (1)

Where Km is classically referred from undergraduate textbook biology as the “affinity” for Na^+^. Because there is a transport mechanism, external Na^+^ must bind, be translocated and then released upon proton exchange in the other direction. As NHE1 function is dictated by the thermodynamic gradients, the direction of transport is also fully reversible upon the respective Na^+^ gradients on each side of the membrane. Consequently, we can write the following kinetic scheme in which every step can operate in each direction.


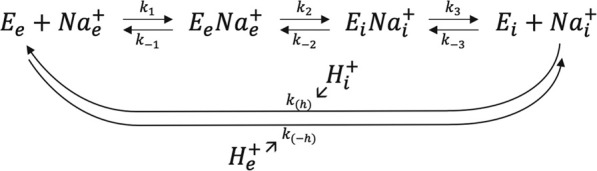


where the symbol (e) represents extracellular and (i) intracellular. For the sake of simplicity, k(h) and k(-h) represent apparent transport rates for H^+^ that are too complex to be developed here mathematically as they contain the cooperative binding and transport mechanism for proton (Lacroix et al., 2004).

Such a simplified mechanism was symbolically encoded in the Maxima computer algebra system (https://maxima.sourceforge.io), thus allowing to express each intermediate as a function of [E_o_] (the total quantity of exchanger), $\left[Na_{i}^{+}\right]$ and $\left[Na_{e}^{+}\right]$ , and finally express and simplify the net rate of $\left[Na_{i}^{+}\right]$ evolution that corresponds here to the transporters steady state velocity $\left(V=d\left[Na_{i}^{+}\right]/dt\right)$ . A tight monitoring of the symbolic results let us group the constants in a meaningful way, giving.

$$V=\left[Eo\right]\frac{\Lambda_{in}\left[Na_{e}^{+}\right]-\Lambda_{out}\left[Na_{i}^{+}\right]}{\alpha +\beta \left[Na_{i}^{+}\right]+\gamma \left[Na_{e}^{+}\right]+\delta \left[Na_{i}^{+}\right]\left[Na_{e}^{+}\right]}$$ (2)

with

$$\Lambda_{in}=k_{1}k_{2}k_{3}k_{\left(h\right)}$$

$$\Lambda_{out}=k_{-1}k_{-2}k_{-3}k_{\left(-h\right)}$$

and

$$\alpha =\left[\left(k_{2}+k_{-1}\right)k_{3}+k_{-1}k_{-2}\right]\left(k_{\left(-h\right)}+k_{\left(h\right)}\right)$$

$$\beta =k_{-3}\left[\left(k_{-2}+k_{2}+k_{-1}\right)k_{\left(-h\right)}+k_{-1}k_{-2}\right]$$

$$\gamma =k_{1}\left[\left(k_{3}+k_{-2}+k_{2}\right)k_{\left(h\right)}+k_{2}k_{3}\right]$$

$$\delta =\left(k_{-2}+k_{2}\right)k_{1}k_{-3}$$

Eq. 2 may look rather complex at first but has several interesting built-in features. The numerator is nicely symmetrical with respect to the kinetic constants, to external Na^+^_e_ and internal Na^+^_i_. The minus sign shows that the transport direction depends on the respective Na^+^ concentrations. It also shows how a rise in intracellular Na^+^ concentration will slow down the inward Na^+^/H^+^ exchange.

Eq. 2 shape is also very close to the Michaelis-Menten Eq. 1 as it can be rearranged into Eq. 3 below.

$$V=\left[E_{o}\right]\frac{\left(\frac{\Lambda_{in}}{\left(\gamma +\delta \left[Na_{i}^{+}\right]\right)}\right)\left[Na_{e}^{+}\right]-\left(\frac{\Lambda_{out}}{\left(\gamma +\delta \left[Na_{i}^{+}\right]\right)}\right)\left[Na_{i}^{+}\right]}{\frac{\alpha +\beta \left[Na_{i}^{+}\right]}{\gamma +\delta \left[Na_{i}^{+}\right]}+\left[Na_{e}^{+}\right]}$$ (3)

If we consider that the intracellular Na^+^_i_ concentration is small enough compared to the extracellular Na^+^_e_, we could then simplify Eq. 3 by neglecting Na^+^_i_ into Eq. 4 below.

$$V=\frac{\left(\frac{\Lambda_{in}}{\left(\gamma \right)}\right)\left[E_{o}\right]\left[Na_{e}^{+}\right]}{\frac{\alpha }{\gamma }+\left[Na_{e}^{+}\right]}$$ (4)

Which is the Michaelis-Menten Eq. 1 for Na^+^_e_ where $Vmax=\frac{\Lambda_{in}}{\gamma }\left[E_{o}\right]$ and $Km=\frac{\alpha }{\gamma }$ .

This implies that when a mutation causes a change in Km, it is risky to assign this to a change in extracellular Na^+^ binding, because its effect could also originate from a change in the off constant (k_3_) for intracellular sodium or more subtly by a change in the k_2_ and or k_-2_ kinetic constants of the ion translocation itself. Such intricate effects are in principle impossible to tease out from steady state measurements because as it can be seen in such equations, the apparent constants have a complex shape and there are more unknown constants than measurable values.

Another important point which deserves attention concerns the inhibitory constants of the NHE acylguanidine inhibitors such as amiloride or cariporide, because they compete with extracellular sodium. Reciprocally Na^+^ also makes competition with the inhibitors and as a consequence, the Michaelis Menten rate equation for transport turns into:

$$V=Vmax\frac{\left[Na_{e}^{+}\right]}{Km\left(1+\frac{\left[I\right]}{K_{i}}\right)+\left[Na_{e}^{+}\right]}$$

**FIGURE 5:**
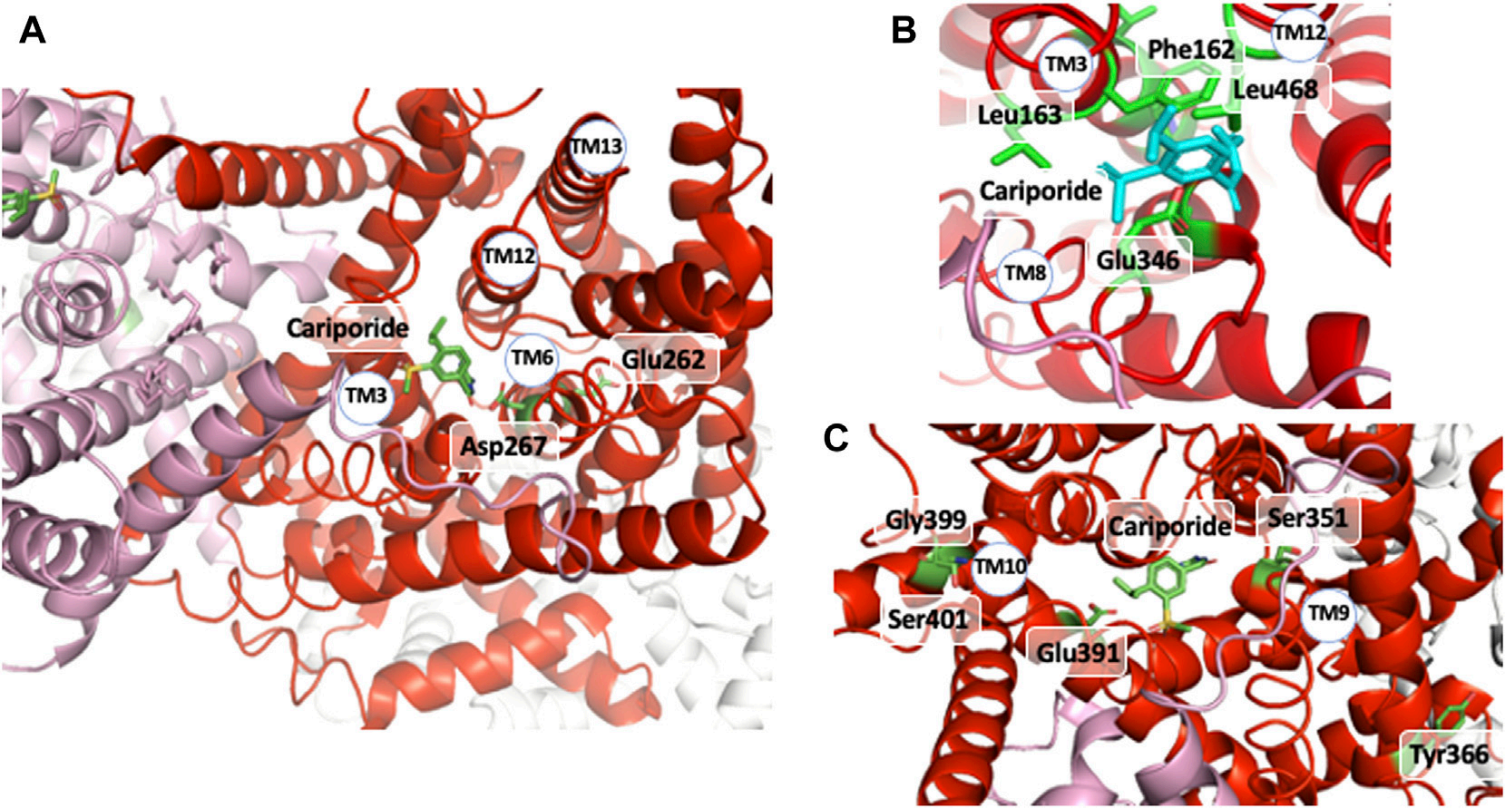
The cariporide-cation binding site: **(A)** upper view of cariporide binding site with the two Glu 262 and Asp 267 critical amino acids for ion translocation underneath. **(B)** Enlarged view of the cariporide/extracellular binding site with the different amino acids shown to be involved directly in this interaction. **(C)** MTS accessible amino acids the mutation of which into Cystein followed by covalent modification affects inhibitor and/or Na^+^ interaction.

Here [I] is the inhibitor concentration, *K*_*i*_ the inhibitor dissociation constant (a true Kd on this occasion) and where *Vmax* and *Km* can have the previous complex expressions. The following facts have to be stressed: 1) a measured K0.5 value cannot be assigned to a *Ki* value because of the presence of *Na* ^*+*^_*e*_, 2) published work reports different K0.5 for the same inhibitor depending on the extracellular cation concentrations used for the measurements, and 3) in case a mutation changes the dose response curve of an inhibitor, the *Km* for *Na* ^*+*^ has to be always measured and a data analysis has to be performed to calculate the actual *K*_*i*_ value if *Km* is found changed. Taken together, these considerations show that transport data can yield the *K*_*i*_ values for inhibitors when properly treated. However, the situation is problematic if not unusable for Na^+^ binding and transport as it is impossible to predict whether a particular position in an NHE is involved in the direct coordination or in the transport of Na^+^ or both. In this respect, it is amazing that most of the residues and positions identified by structure-function studies fall in places that belong to these categories in NHE1 3D structure as the structural complexity and organization now offers insight that could not be revealed by placing the identified crucial amino acids within the previous topological models. Indeed, a constellation of side chains atoms is remarkably in the right place to directly interact with the cariporide structure. As this inhibitor contains a guanidine moiety with a structure similar to a partly hydrated Na^+^, the NHE1 structure also reveals how this cation could sit in its external binding pocket (Figure 5). Those correspond to 1) Leu 163 and Phe162 in the remarkable VFFLFLLPPII TM3 sequence (Counillon et al., 1993) (Counillon et al., 1997) (Touret et al., 2001) that makes a very beautiful π−stack with the inhibitors’ aromatic ring (2.6 Å distance), 2) TM8 Glu346 that is at less than 2.8 Å from the guanidine group (Khadilkar et al., 2001; Noël et al., 2003). Of note a recent article by Fliegel’s group highlighted Leu468 of TM11 that is also in a very close position to the hydrophobic 5- substituents of cariporide, thereby providing a molecular basis on the mechanism by which an increased hydrophobicity of these inhibitors’ groups can decrease their Ki values by two orders of magnitude (Li et al., 2021). Finally, the absence of side chain of Gly352 that lies underneath the inhibitor appears to be a steric effect as an amino acid with a large side-chain would collide with the inhibitor (Khadilkar et al., 2001).

In a recent work, Jinadasa et al. (2015) used cysteine substitution and MTS accessibility to map amino acids involved in the interaction with ethylisopropylamiloride, a molecule close to cariporide. This revealed interesting candidates as shown in Figure 5C, clearly accessible to externally-applied MTS, but more likely to exert some distance effect on inhibitor or Na^+^ interaction.

Apart from these residues that are obviously in direct interaction with the competitive inhibitor and very likely with the partially hydrated transported Na^+^, mutations at some other positions appear to have indirect distant effects. As explained above in the kinetic analysis of NHE1 transport, mutations that modify Km or *Ki* values can have conformational effects that impact kinetic steps instead of binding. When far away but in the same plane as cariporide they likely exert such conformational domino effects on the structure, such as TM11 His 473, Met 476 (Li et al., 2021) or TM2/EL2 Gly 148; and Phe155, or His 349 (Orlowski and Kandasamy, 1996; Khadilkar et al., 2001). Similarly, we described mutations of amino acids in the TM3 sequence, that affected the Km and apparent inhibition constants of competitors by such distance conformational effects. Those were in particular Gly 174 (Counillon et al., 1997), Ile 169 and 170, the mutation of which were able to revert the Phe162Ser mutation (Touret et al., 2001). From the structure, those are indeed far from the external binding site, thereby confirming the difference between binding and kinetic effects.

### The Cooperative Regulation of Transport by Intracellular H^+^

Positive cooperativity demarks from classical Michaelis-Menten saturation as the hyperbolic shape of the dose-response curve is bent to yield a S shape curve, termed sigmoid. Being steeper than a classical hyperbolic, the cooperative response often constitutes a molecular switch, the setpoint of which can be modified by the interaction with different allosteric modulators. Hence, most enzymes or transporters possessing a cooperative behavior have been selected by evolution at strategic positions to activate or block critical biochemical pathways. As protons are one of the most, if not the most relevant ions in physiology, evolution has firmly incorporated cooperativity in NHEs kinetics. Technically, it must be noted that using pH as a variable can be misleading at first glance, because it is the cologarithm of the actual free H^+^ ion concentration that is the relevant parameter, with sub millimolar concentrations between 10^–7^ and 10^–8^ mol/L in the cytosol in physiological conditions. This is particularly important for graphical representations as the Log scale that is embedded in pH representations can be confusing when analyzing possible cooperativity.

Mathematically, sigmoidal equations rates for enzymes or transporters are different from the logistic equation often used to fit sigmoids and whatever the mechanism, can be written as a fraction of two polynomials for the substrate. This stems from the fact that proteins displaying cooperativity possess more than one binding site for their substrate, leading to multiple equilibria. This means that the reaction order is superior to one for the substrate, and this translates in exponents superior to one for its concentration in the kinetic equations. Another important feature is that those binding sites are neither identical nor independent, meaning that cooperative proteins exist in different conformational states bearing differences in substrate binding or transport. Beyond these general features it is challenging to precisely decipher the underlying mechanisms for any cooperative behavior, because a near infinity of polynomial fractions can fit the same sigmoid. As each equation corresponds to a distinct possible process, it means that it is in principle conceivable to hypothesize an infinite number of mechanisms that would all fit, provided that the constants (that can be fairly complex as explained previously) and exponents are well adjusted. Hence to discriminate between the potential models, extensive information about mutagenesis and/or structure is needed in addition to kinetics and dose responses. Because the required formalism may go beyond the purpose of this review, the interested reader is encouraged to consult advanced enzyme kinetics textbooks such as Bisswanger (2008).

The above-mentioned findings have led to interesting questions concerning the allosteric regulation of NHEs by intracellular H^+^. The first main mechanism proposed after the discovery of the NHEs cooperativity, was that of a monomer with a “proton sensing site” that would allosterically regulate the affinity of the transport site. This led many colleagues to try to identify such a sensor by mutating candidate amino acids, mostly histidines, the pKa of which allows them in principle to bind/unbind H^+^ around pH7. However, without entering in mathematical details, a cooperativity model resulting from a mechanism involving a simple binding of an alternative H^+^ in a non-transport site would unsatisfactorily fit the data due to the reciprocal dependency of the transport and sensor sites (Lacroix et al., 2004). Considering the NHEs dimeric structure and a combination of mutagenesis and kinetic analyses we proposed a mechanism in which the two protomers, strongly interlocked within a symmetrical dimer, would oscillate between a low and a high affinity state for H^+^ in a concerted manner (Monod et al., 1965). Any change that would affect the balance between these two forms, from covalent modification of the protein to its interaction with different molecules or ions, would change the sigmoidal shape and therefore provide H^+^ sensing, without the need for an additional binding site. This is largely mediated through the C-terminal region of NHE1 that contains multiple regulatory sites for signaling pathways. Depending on its interactions with lipids, ATP, proteins and on the balance between its multiple phosphorylations and dephosphorylations (Hendus-Altenburger et al., 2016; Hendus-Altenburger et al., 2019), this region can modify NHE1 cooperativity for protons. In particular, the structure shows that the Pro571-Ser591 short helical antiparallel dimer could be instrumental in this allosteric coupling.

Such regulatory mechanism provides the selective advantage to operate as a sensitive coincidence detector for infinite combinations of stimuli, an action that would be impossible to achieve by direct modification of a proton regulator site.

**FIGURE 6:**
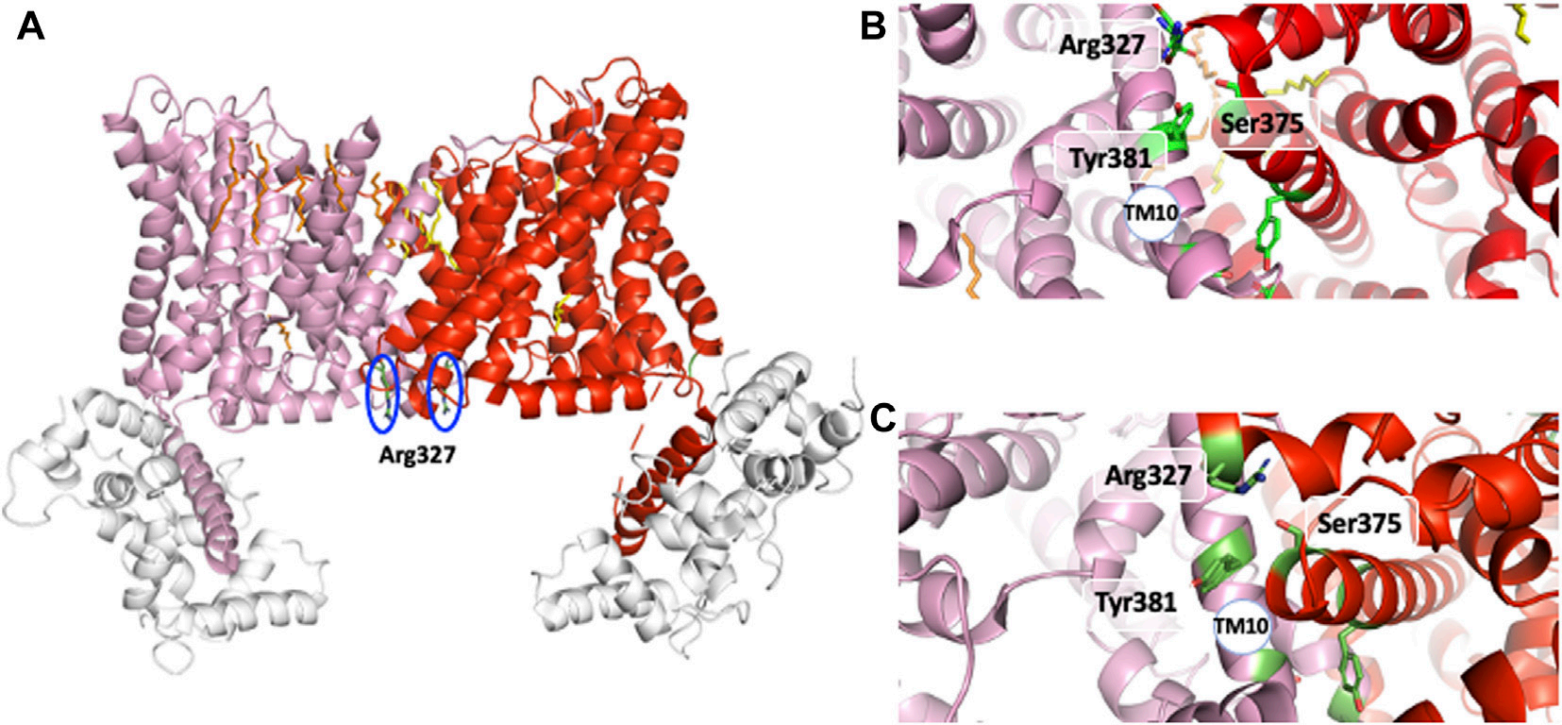
The interfacial amino acids that govern the NHE1 allosteric transition. **(A)** the two Arg327 at the dimer interface in the main pH 6.5 structure. **(B)** The Arg 327, Ser 375, Tyr 381 triad at the dimer interface at pH6.5. **(C)**The Arg 327 Ser 375, Tyr 381 triad at the dimer interface in the amiloride bound outward-facing NHE1. See the alpha helical boundary change and the changes in orientation between the two structures.

**FIGURE 7:**
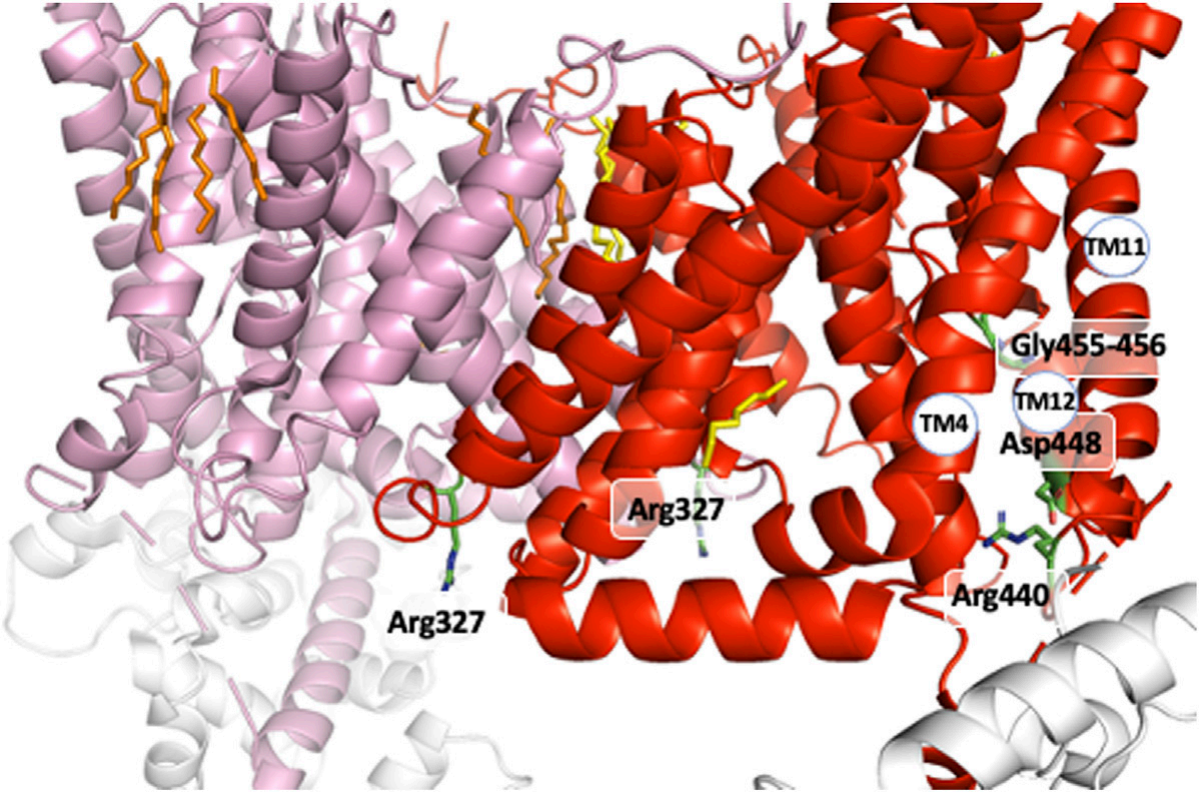
The other positions that govern the NHE1 allosteric transition for H^+^. The two Arg 440 and Asp 448 close to the C terminal intracellular region and the Gly 455-Gly 456 amino acids in the interrupted alpha helix of TM11.

A strong support for this mechanism came from the identification of distinct mutations that locked NHE1 in non-cooperative conformation and/or the existence of NHEs with lost cooperativity for protons. A similar low affinity non cooperative NHE1 could be obtained respectively through the mutation of conserved Arginines 327 (Lacroix et al., 2004) and 440 (Wakabayashi et al., 2003a; Wakabayashi et al., 2003b), Serine 375 and Tyrosine 381 (Hisamitsu et al., 2006). Interestingly, total (Lacroix et al., 2004) or partial NHE1 C-terminal truncation, such as the above-mentioned Met 561-Ala575 sequence, yielded the same low affinity non-cooperative NHE1 (visible when plotted as a function of H^+^ instead of pH in Hisamitsu et al., 2004). Interestingly, Arg 327 is not conserved in the non-cooperative NHE7 that displays a high affinity for intracellular H^+^ (Milosavljevic et al., 2014). As arginine’s pKa is 12.5, those amino acids are not good candidates for direct H^+^ binding and release at physiological pH values. In contrast, bearing a positive charged group at the end of a flexible arm could be extremely useful either to couple to other amino acids or act as a short probes for the electrostatics of their environment. Similarly, the Ser 375 and Tyr 381 are not deprotonable at physiological values but may form hydrogen bond and exert a conformational role. Considering the effects of all these mutations, we can predict that these residues must be placed at strategic positions within the dimer. Indeed, the analysis of the NHE1 structure in different conditions reveals a critical position for the two Arg327 that are situated right at the interface between the two protomers (Figure 6A). In the pH6.5 structure (Figure 6A), these two arginines point the positive extremity of their side chains towards the cytosol, and could work exactly as the previously discussed electrostatic probes. Even more strikingly, Arg 327 is in very close vicinity of Ser 375 and Tyr 381, the two critical residues previously mapped in the dimer interface (see above) by the Wakabayashi group. In the 6.5 structure, the main backbone carbonyl of Arg 327, and the hydrogens of Ser 375 and Tyr 381 side chain hydroxyl groups are at optimal distances (∼3 Å) to hydrogen bond. Ser 375 substitution into a cysteine led to a low affinity of NHE1 for protons and this had been interpreted as a possible effect of disulfide bond crosslinking that would block the structure. However, Figure 4B shows that these two side chains are pointing in an opposite direction making disulfide bond formation unlikely. Interestingly, the cariporide-bound structure shows a totally different configuration in which Arg 327 is not in a flexible loop. Indeed, its main chain carbonyl is engaged in an alpha helix, with no possibility to interact with the previous amino acids, its side chain being 6–8 Å apart from the Ser 375 hydroxyl. Moreover, in this conformation, the Tyr 381 side chain is now totally opposite to the dimer interface. Taken together, the input from the structural information highlights the importance of the previously discovered amino acids in a symmetrical dimer. In addition, the conformational changes resulting in the exquisite sensitivity of NHE1 for protons (Lacroix et al., 2004) are uncovered. Arg 440 mutations were also identified for giving a very similar phenotype as those of Arg 327 while Asp 448, Gly 455, and 456 mutations resulted in an enhanced sensitivity to protons. These positions in the structure are far away from the dimer interface. Gly 455 and 456 are in the TM11 non-helical region (Figure 7). Arg 440 and Asp 448 are present in a perfect pocket constituted by helices TM4, 11, 12, and the CHP binding region (Figure 7). Interestingly, mutations that affect NHE1 activity or its response to intracellular pH have been identified between Leu 432 and Lys 443 by the Fliegel’s group (Wong et al., 2019).

It is important to stress that all the mutations that decrease the H^+^ sensing for intracellular H^+^ involve amino acids that cannot be protonated/deprotonated around the NHE1 setpoint for intracellular H^+^, nearby physiological pH. In contrast, they are all at interfacial positions, either at the dimer interface, or at the boundary with the regulatory C-terminal loop or at hinge sequences of NHE1. Another important point is that all the Cryo-EM structures correspond to symmetrical dimers, and not to different conformations within the same dimer. Taken in aggregate, the above summarized studies provide a strong accumulation of results in favor of a concerted cooperative mechanism for proton sensing.

## Pending Questions: Reversibility and Stoichiometry

**FIGURE 8:**
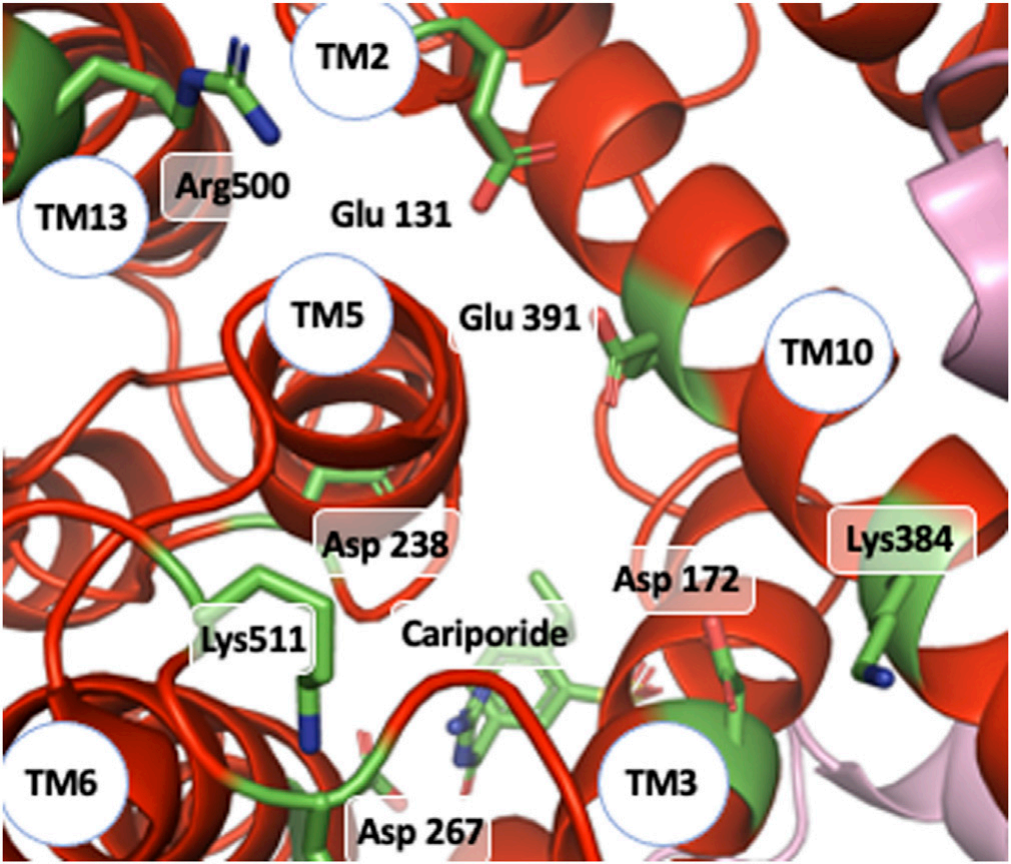
NHE1 reverse exchange: view of the funnel on the intracellular side of the cariporide-bound structure and amino acids that could be involved intracellular Na^+^ coordination as well as possible H^+^ binding and release.

As mentioned in a previous section of this manuscript, NHE1 is a reversible transporter. This implies some symmetry in the distribution of protonable amino acids that could bind Na^+^ and/or H^+^ within its structure. Indeed, opposite to the cariporide binding site there is a large funnel opening to the intracellular side. Very good candidates such as Glu 131, Asp 172, or Asp 238 (Figure 8) are present in this region and could very well coordinate Na^+^ as proposed by (Dong et al., 2021), taking into account the structure homology with the Thallium bound PaNhaP structure. Other amino acids such as Glu 391, Lys 511 or Arg 500 could also participate in shaping the electrostatics of this funnel (Figure 8). The critical Asp 267 is situated at a pivotal position between the outward facing funnel that bears the cariporide binding site (see Figure 8) and this inward facing funnel.

Taken together what emerges from this model is that a change of electrostatics due to Na^+^ ion binding in one of these funnels could trigger a reversible conformational change that then would cause Na^+^ translocation towards the other funnel.

In such a mechanism, Na^+^ and H^+^ binding on the previously mentioned carboxylic groups could be mutually exclusive. Such a feature would therefore constitute a molecular basis for the antiport function of NHE1. One important question that remains unanswered yet is the molecular basis for the 1:1 exchange stoichiometry. This is a non-trivial question for at least two reasons: firstly, the proton is the smallest possible cation, and therefore it could bind and unbind different side chains, hydrogen bond, or cross energy barriers to travel through rigid sections of the protein by other mechanisms such as quantum tunnelling effect, like in ice (Atkins and de Paula, 2013) or enzymes (Bothma et al., 2010). Secondly, one must not forget that this 1:1 stoechiometry is a macroscopic feature of the transport, that is measured on a large quantity of molecules and transport cycles. Therefore, it cannot be excluded that different microscopic transport mechanisms averaging to 1:1 could coexist. Solving this fascinating problem will likely require a combination of mutagenesis, coupled to sophisticated kinetics and molecular simulations.

### Take Home Messages

As stated in the introduction, the structural determination of NHE1 and NHE9 have provided a long-awaited and decisive breakthrough in the understanding of the Na^+^/H^+^ exchange world. The aim of our short review in the context of this dedicated issue was to put in a historical perspective some of the main findings that shaped the NHE1 knowledge for many years, namely the topology, dimeric structure, biochemical analysis and key mutations. Learning from the past and considering the advances already made in the field, it is obvious that further progress is contingent on combining structural information with data from functional measurements with the adequate methods that will have to use more sophisticated mathematics and modeling. Future progress will also come from setting up more resolutive measurement methods such as extremely fast presteady state kinetics.

Finally, while the unraveling of the tridimensional structure of the Na^+^/H^+^ exchanger is clearly a spectacular leap forward in the field, it is gratifying for the pioneers in this research area that most of their vision on their favored molecule has stood the test of time. In the meantime, it is fascinating to see how results that could appear rather abstract are now highlighted in a very visual and even aesthetic perspective in those structures. Times are changing -so are models but for both the former and the current Na^+^/H^+^ exchanger scientists “beauty is in the eyes of the beholder” (Aronson et al., 1982; Wakabayashi et al., 2003b).
